# Relating Anthropometric Profile to Countermovement Jump Performance and External Match Load in Mexican National Team Soccer Players: An Exploratory Study

**DOI:** 10.3390/sports13070236

**Published:** 2025-07-18

**Authors:** Israel Rios-Limas, Carlos Abraham Herrera-Amante, Wiliam Carvajal-Veitía, Rodrigo Yáñez-Sepúlveda, César Iván Ayala-Guzmán, Luis Ortiz-Hernández, Andrés López-Sagarra, Pol Lorente-Solá, José Francisco López-Gil

**Affiliations:** 1 Sports Nutrition Department, Mexican Football Federation (FMF), Mexico City 50200, Mexico; irios@fmf.mx; 2 Ibero-American Network of Researchers in Applied Anthropometry (RIBA), 04120 Almería, Spain; carlos.amante@academicos.udg.mx (C.A.H.-A.); wiliamcarvajal790@gmail.com (W.C.-V.); cesar.ayala@ibero.mx (C.I.A.-G.); 3 Nutritional Assessment and Nutritional Care Laboratory (LECEN), Division of Health Sciences, Tonala University Center, University of Guadalajara, Tonalá 45425, Mexico; 4 Subdirectorate of Teaching and Research, Institute of Sports Medicine (IMD), Havana 10800, Cuba; 5 Faculty of Education and Social Sciences, Universidad Andres Bello, Viña del Mar 252000, Chile; rodrigo.yanez.s@unab.cl; 6 Health Department, Iberoamerican University, Mexico City 01219, Mexico; 7 Nutrition and Physical Activity Laboratory, Metropolitan Autonomous University Campus Xochimilco, Mexico City 04960, Mexico; lortiz@correo.xoc.uam.mx; 8 Sports Science Department, Mexican Football Federation (FMF), Mexico City 50200, Mexico; anlopez@fmf.mx; 9 Mexico National Team, Mexican Football Federation (FMF), Mexico City 50200, Mexico; plorente@fmf.mx; 10 School of Medicine, Universidad Espíritu Santo, Samborondón 092301, Ecuador; 11 Vicerrectoría de Investigación y Postgrado, Universidad de Los Lagos, Osorno 5311269, Chile

**Keywords:** soccer, anthropometry, body composition, sports nutritional sciences, athletic performance

## Abstract

***Background/Objectives:*** The role of body composition in sports performance has been widely studied, particularly in soccer. Understanding how anthropometric characteristics impact movement efficiency and neuromuscular performance is crucial for optimizing player performance. This study examined the relationship between body composition and locomotor performance in elite soccer players. ***Methods:*** Thirty-six male soccer players from the Mexican National Team participated in the study, where body composition was assessed using surface anthropometry. Players underwent tests to measure countermovement jump (CMJ) performance, sprinting speed, maximum acceleration, and distance covered during two games of the CONCACAF Nations League quarterfinals. Correlation matrices were created to identify the most significant associations, followed by generalized linear models (GLMs) to associate body composition variables with performance metrics. ***Results:*** Anthropometric profile tables were created by playing position. Higher body fat percentage (%BF) was associated with lower performance. Specifically, higher %BF was associated with slower sprint speed (B = −0.74 m/s, *p* < 0.001) and shorter distance covered (B = −4.86 m/min, *p* < 0.001). Conversely, greater muscularity, reflected by corrected girth values for the thigh and calf, was associated with improved CMJ performance. Thigh corrected girth was positively associated with concentric mean force (B = 48.85 N, *p* < 0.001), and calf corrected girth was positively associated with peak power (B = 240.50 W, *p* < 0.001). These findings underscore the importance of low body fat and high lean mass for efficient movement. ***Conclusions:*** The results highlight the critical role of body composition in enhancing soccer performance, particularly for explosive movements like jumps, sprints, and accelerations. This study suggests that monitoring and optimizing body composition should be a central focus of nutrition, training, and conditioning strategies, adapted to the specific positional demands of professional soccer.

## 1. Introduction

The anthropometric and body composition profiles of soccer players have been widely reported across Latin American [1,2,3,4] and European samples [5,6,7], from youth to elite levels. These studies consistently report differences by playing position or age group. For instance, Rodríguez-Rodríguez et al. [1] and Junior et al. [2] observed that goalkeepers tend to be taller and heavier, while forwards generally present greater muscle mass. Similarly, Holway et al. [3] and Toselli et al. [5] noted that older players exhibit higher body mass, muscle mass, and bone/muscle index compared to younger counterparts. While these studies provide a comprehensive anthropometric description, to the best of our knowledge, few have directly examined the relationship between these characteristics and performance indicators such as match load or neuromuscular capacity. One exception is the work by Radzimiński et al. [6], which highlighted the relevance of further exploring how anthropometric factors may influence physical performance in competitive settings.

This gap is particularly relevant given that elite soccer is characterized by the complex interaction of physical, physiological, tactical, and contextual variables that, together, determine on-field performance. International studies developed with youth [8,9,10], semi-professional [11], intercollegiate [12], and professional female [13] soccer players have made significant progress in characterizing these variables. These studies reported that anthropometric parameters, body composition, and biomechanics directly influence key aspects such as sprints, vertical jumps, direction changes, power, explosive strength, metabolic capacity, and flexibility. However, the aforementioned findings were primarily observed in non-elite or female samples, and few studies have been conducted with elite soccer players [5,6]. Hence, despite these advances, many investigations lack an integrative approach incorporating indicators of functional and contextual performance, principally in elite male soccer players or during official matches. This limits a comprehensive understanding of the interactions between body structure and function.

Technologies such as the Global Positioning System (GPS) have proven essential for evaluating physical performance during training and competition. Previous studies have used this technology to analyze metrics such as total distance covered, high-intensity running, accelerations, and decelerations, as well as estimations of energy expenditure during exercise, providing a detailed and objective perspective of performance on the pitch [14,15,16,17,18]. Nevertheless, the relationship between body composition and speed, distance, and acceleration has not been explored in elite soccer players. In young Chilean professional soccer players [9], greater muscle mass in the lower limbs and lower body fat percentages have been associated with improved acceleration and sprint performance. In professional European soccer players [6], high-speed running distance was negatively correlated with lipid mass. In professional Greek soccer players [18], speed running was negatively related to stretch stature and body mass. In Spanish semi-professional soccer players [11], it was reported that players have morphologically distinct profiles according to playing position, which, in turn, correlates with speed, distance covered, squat jump, countermovement jump, and maximum oxygen uptake. These findings illustrate how body composition and anthropometrics relate to the competitive and positional demands of the match in non-elite athletes. Also, these findings underscore the need for further research considering a broader range of factors and their interactions with performance in elite soccer players.

International tournament data provide further context for highly trained athletes. Ju et al. [19] reported that, although no significant differences in high-intensity running distances exist between confederations such as CONCACAF, UEFA, and CONMEBOL, European players tend to outperform others in technical–tactical metrics, such as passing accuracy and line-breaking actions.

Mexico’s case is especially relevant in this global context, as it is one of the top-performing teams in the CONCACAF region. Despite the recent success of the Mexican National Team—champions of the 2025 CONCACAF Nations League. The studies conducted with Mexican soccer players stem from descriptive investigations of youth [20], first division plus under 17, and under 20 [21] soccer players. Only one study was performed with Mexican National Team soccer players [4], in which anthropometric and performance indicators were studied but not correlated. Therefore, research that comprehensively addresses Mexico’s club and national team’s physical, physiological, and morphofunctional aspects is scarce, limiting our understanding of how these variables interact and influence performance.

This lack of integrative information has hindered the development of evidence-based training and monitoring strategies tailored to the specific characteristics of Mexican soccer players. Therefore, exploring how physical, physiological, and morphofunctional variables interact with match performance is essential to optimize sports professionals’ decision-making on and off the field.

In this context, a comprehensive analysis of anthropometric, countermovement jump, and match performance variables can provide deeper insight into soccer success. Accordingly, this study addresses this knowledge gap by characterizing and analyzing the multidimensional relationships between these variables in elite Mexican soccer players. This will be useful in advancing scientific understanding and providing practical knowledge to enhance players’ individual and collective development in an increasingly demanding competitive environment.

## 2. Materials and Methods

### 2.1. Study Design

A descriptive, cross-sectional study was conducted. Participants attended the assessment location once to collect anthropometric and countermovement jump test data. Additionally, before two FIFA match dates, players underwent performance monitoring assessments. The study followed the Strengthening Reporting of Observational Studies in Epidemiology (STROBE) guidelines [22,23].

### 2.2. Setting

The study was conducted during the Mexican National Soccer Team’s training camp on the FIFA date heading into the Concacaf Nations League quarterfinals. The training camps took place from November 11 to 19 2024: from November 11 to 15 in San Pedro Sula, Honduras, and from November 16 to 19 at the Mexican Football Federation’s (FMF) High-Performance Center (CAR, by its acronym in Spanish) in Mexico City.

The study was approved by the Research Ethics Committee of the Autonomous Metropolitan University Campus Xochimilco (code: DCBS.CD.071.21) and was registered at clinicaltrials.gov (identifier: NCT06941064). Individual information (i.e., demographic, descriptive data, and sports experience) was collected using a brief form for practical sports nutrition advice [24]. All participants provided verbal informed consent after the principal investigator thoroughly explained the study’s objectives, procedures, benefits, and potential risks. Written consent could not be obtained due to personal and contractual restrictions associated with their professional clubs. Nevertheless, according to Winter and Maughan [25], the principles of informed consent may not be fully upheld in professional sports, principally when data are collected from routine measurements taken at various stages of the competition and during the off-season. However, this study was conducted using the ethical principles established in the Declaration of Helsinki [26] and international guidelines for Good Clinical Practice.

### 2.3. Participants

Thirty-six male soccer players from the Mexican National Team participated in the study (age: 27.3 years old [IQR: 24.2–28.4]; stretch stature: 177.9 cm [IQR: 171.3–182.9]; body mass: 77.9 kg [IQR: 71.3–82.9]; competitive sports experience: 10.4 years [IQR: 7.2–13.6]; weekly training volume: 20 h [IQR: 16.6–23.4]). All participants were members of the Mexican National Team at the time of the study and had competed in international tournaments. Specifically, 11 players (30.6%) were playing for international clubs, including England (*n* = 3; 8.3%), Italy (*n* = 2; 5.6%), Russia (*n* = 2; 5.6%), Belgium (*n* = 1; 2.8%), Greece (*n* = 1; 2.8%), Portugal (*n* = 1; 2.8%), Saudi Arabia (*n* = 1; 2.8%), and Denmark (*n* = 1; 2.8%). The remaining 25 players (69.4%) were participating in Mexico’s Liga MX. Additionally, 44% of the players had competed in at least one FIFA World Cup prior to the assessment date. To determine the appropriate sample size for detecting a strong association between body composition and GPS-derived performance metrics in elite football athletes, we conducted an a priori power analysis using G*Power (version 3.1.9.7). Assuming a large effect size (Pearson’s correlation coefficient, *r* = 0.80), a significance level (α) of 0.05, and a desired power (1 − *β*) of 0.80, the analysis indicated that a minimum of 25 participants would be required to detect a statistically significant correlation. This sample size is feasible within the context of a national football team cohort and ensures sufficient power to validate the hypothesized association.

**Figure 1:**
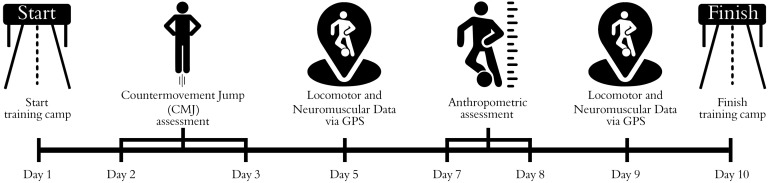
Timeline of assessments conducted during the 10-day training camp. The training camp began on Day 1. Countermovement jump (CMJ) assessments were conducted on Days 2 and 3 in San Pedro Sula, Honduras. Locomotor and neuromuscular data were collected via GPS during match play on Day 5 (Game 1, San Pedro Sula) and Day 9 (Game 2, Toluca, Mexico). Anthropometric assessments were performed on Days 7 and 8 at the High-Performance Center (CAR FMF), Mexico City. The camp concluded on Day 10. All assessments were scheduled to avoid interference with training and to ensure data quality and consistency.

Athletes who attended the assessment area during the training camps were invited to participate in the study, and those who gave verbal assent were included in the sample. Players not called up to the Mexican National Team on the above dates were excluded from the study. The timeline of the evaluations carried out is shown in Figure 1.

### 2.4. Assessments

#### 2.4.1. Anthropometric Measurements

Anthropometric measurements were performed by a Level 2 ISAK-certified anthropometrist, following the International Society for the Advancement of Kinanthropometry (ISAK) standards [27]. Body mass (kg) was measured using a digital scale with a precision of 50 g (SECA^®^ 874, Hamburg, Germany). Stretch stature (cm) was assessed using a portable stadiometer with a precision of 1 mm (SECA^®^ 217, Hamburg, Germany). Girth measurements (cm) were obtained using a non-extensible, flexible steel tape with a precision of 1 mm (Rosscraft^®^, Surrey, Canada). Skinfold thicknesses (mm) were measured with a skinfold caliper accurate to 1 mm (Harpenden, Holtain Ltd., Bryberian, Crymmych, Pembrokeshire). All instruments were calibrated before the measurement process.

All anthropometric variables were measured at least twice. A third measurement was taken if the difference between the first and second measurements exceeded 5% for skinfolds or 1% for any other variable. The mean value was used for data analysis when two measurements were taken. The median value was used for data analysis when three measurements were taken [27]. Intra-evaluator technical error of measurement (TEM) was calculated (25), yielding 4.06% for skinfolds and 0.89% for other variables.

#### 2.4.2. Body Composition

Body composition was estimated using surface anthropometry. Body fat percentage (%BF), specifically lipid mass, was calculated using five predictive equations: Yuhasz [28], Sloan [29], Wilmore and Behnke [30], Durnin and Womersley [31], and Jackson and Pollock [32]. Adipose mass was then derived using the Ross and Kerr equation [33]. Meanwhile, muscle mass (MM) was estimated using two anthropometric equations: Martin et al. [34] and Lee et al. [35].

Additionally, adiposity and muscularity were analyzed using skinfold sums and corrected girths, respectively. The sum of six skin folds consisted of the triceps, subscapular, supraspinal, abdominal, thigh, and calf skinfolds. Meanwhile, the eight skinfolds’ sum included the triceps, subscapular, biceps, iliac crest, supraspinal, abdominal, thigh, and calf skinfolds. Corrected girths were calculated for the arm, chest, thigh, and calf using the following equation: girth in centimeters − (π × skinfold in centimeters) [36]. Triceps, subscapular, anterior thigh, and calf skinfold were used to compute corrected girths. The sum of the four corrected girths was calculated.

#### 2.4.3. Countermovement Jump

The countermovement jump (CMJ) assessments were conducted using a ForceDecks Max platform (VALD Performance, Brisbane, Australia). This allows for a detailed kinetic and temporal variables analysis through a dual high-precision platform system [37]. The assessment protocol has been previously standardized and validated [38].

After a brief warm-up, including dynamic stretches and sub-maximal jumps (five individual repetitions and two sets of five repeated CMJs), participants performed three maximum-effort CMJs at a self-selected counter-movement depth, with approximately one-minute rest intervals between each attempt. During each jump, participants were instructed to “jump as fast and as high as they could”, keeping their hands on their hips. This instruction was standardized, as it influences the force-time characteristics of the CMJ.

Force data were collected at 1000 Hz and processed to obtain variables such as take-off velocity, jump height (calculated from the take-off velocity), and the modified reactive strength index (jump height divided by time to take-off). The moment of the jump was calculated as the product of take-off velocity and body mass. The average of the three attempts was used for statistical analysis.

#### 2.4.4. Locomotor and Neuromuscular Data

Locomotor and neuromuscular external load metrics were obtained using WIMU Pro GPS tracking units operating at 10 Hz (RealTrack Systems, Almería, Spain). This system has been validated for time–motion analysis in soccer, demonstrating strong intra- and inter-unit reliability (mean velocity bias ranging from 1.18 to 1.32 km·h^−1^; mean distance bias between 2.32 and 4.32 m; intraclass correlation coefficient > 0.93) [39]. To minimize the variability between units, each player was assigned a specific device that they used consistently throughout the data collection period. The GPS units were positioned in a vest pocket designed explicitly by the manufacturer. The devices were powered on 15 min before kickoff to ensure optimal data capture.

On average, 12.1 ± 2.4 satellites were connected during recordings, and the horizontal geometric dilution of precision was 0.57 ± 0.14—both values reflecting a high-quality GPS signal [40,41]. After each match, data were downloaded using SPro software (version 980, RealTrack Systems, Almería, Spain). All locomotor and neuromuscular variables were adjusted relative to the total match duration. The thresholds used for high-speed running and high metabolic load were >21 km·h^−1^ and >25.5 W·kg^−1^, respectively. The variables included in the analysis were distance per minute (Distance [m/min]), maximum speed (Max speed [km/h]), number of accelerations ≥ 3 m·s^−2^ per minute (Acc+3/min), number of decelerations ≥ 3 m·s^−2^ per minute (Dec+3/min), high metabolic load distance per minute (HMLD/min), sprint distance per minute (Sprint/m), number of sprints per minute (N Sprints/m), and high-speed running per minute (HSR/min).

### 2.5. Study Size

A non-probabilistic convenience sampling method was used. All participants were members of the Mexican National Team at the time of the study and had competed in international tournaments. Notably, 44% had participated in at least one FIFA World Cup, and 29.7% were playing for European soccer clubs. Therefore, the sample represents Mexican elite soccer players. Moreover, according to Pituch and Stevens [42], a minimum of 15 participants is sufficient for developing reliable equation models, supporting the adequacy of the current sample size.

### 2.6. Statistical Methods

Statistical procedures were performed using R version 4.4.1 (R Core Team, Vienna, Austria) within the integrated development environment RStudio version 2023.06.2+561 “Mountain Hydrangea” (Posit, PBC, Boston, MA, USA).

The age, anthropometric, and body composition variables and indexes had asymmetric distributions, so the median and interquartile range (IQR) were used to describe these characteristics (Table 1). After a Kruskal–Wallis test, statistical differences between playing positions were assessed using Dunn’s post hoc test with Bonferroni correction.

Due to the nature of the competition, not all participants who underwent anthropometric assessment could participate in one or both matches. This meant that the sample size differed for some analyses. Table 1 shows the information for the overall sample (*n* = 36), while, for subsequent analyses, only the data from active athletes during competitions were used (*n* = 22).

A general exploratory correlation matrix was constructed to identify relationships among all study variables. Subsequently, draftsman plots were generated to summarize and visualize the most relevant and statistically significant correlations.

Based on the previous exploratory correlations, generalized linear models (GLMs) were developed using robust estimation methods, since the dependent variables did not follow a Gaussian distribution. The independent effect of anthropometric variables on physical performance measures, without including moderator variables, was examined with these models. Additionally, Bonferroni correction was applied across the GLMs to account for multiple comparisons. The theoretical model guiding this analytical strategy is expressed as follows:

*Y* = *i*_*Y*_ + *β*_1_*X* + *β*_2_*W* + *ε*_*Y*_

where *Y* represents the dependent variable (e.g., jump height or energy expenditure); *i_Y_* is the regression intercept; *β*_1_ and *β*_2_ are the estimated regression coefficients for the predictor variables; *X* is the main independent variable (e.g., stretch stature, BMI, and skinfolds sum); *W* represents a second predictor variable (not used as a moderator in this study); and *ε_Y_* is the random error term.

It is worth noting that, although the model notation includes two predictors, *X* and *W*, this analysis did not incorporate interaction or moderation effects. Based on previous methodological recommendations, all models were adjusted using robust procedures to account for potential heteroscedasticity and outlier sensitivity. All models were adjusted by the athlete’s age and body mass. Statistical significance was set at *p*-value < 0.05.

To ensure the validity of the GLMs, multicollinearity among the predictor variables was assessed using the Variance Inflation Factor (VIF). Variables with VIF values greater than 5 were carefully examined. Since no variable exceeded this threshold, multicollinearity was not considered a concern, and no further adjustments were required.

## 3. Results

### 3.1. Anthropometric Profile

Table 1 presents the anthropometric characteristics of Mexican soccer players according to their playing position.

Regarding body mass and stretch stature, goalkeepers were significantly heavier and taller than offensive midfielders (*p* < 0.05), while central defenders were also significantly taller and heavier than offensive midfielders (*p* < 0.05).

In terms of girths, goalkeepers showed significantly greater forearm girths compared to offensive midfielders (*p* < 0.05). Central defenders exhibited larger mid-calf and corrected mid-calf girths than offensive midfielders, and forwards also showed significantly higher values in these girths compared to offensive midfielders (*p* < 0.05).

As for muscularity indicators, both central defenders and forwards showed significantly greater values in the sum of four corrected girths (Σ4 C.G) and estimated muscle mass compared to offensive midfielders (*p* < 0.05).

No significant differences were found between playing positions in age; raw skinfold measurements; body mass index; waist-to-hip ratio; sum of skinfolds; body fat percentage; or corrected arm, chest, and thigh girths (*p* > 0.05).

### 3.2. Anthropometric Profile and CMJ Performance

The players exhibited a median jump height of 40.7 cm, with an interquartile range (IQR) of 39.8–43.9 cm. Peak power reached a median of 4434.0 W (IQR: 4028.0–4782.0), while concentric impulse was 220.9 N/s (IQR: 197.6–232.8). The median concentric force was 1619.0 N (IQR: 1531.0–1726.0), and the median concentric power was 2573.0 W (IQR: 2350.0–2679.0).

**Figure 2:**
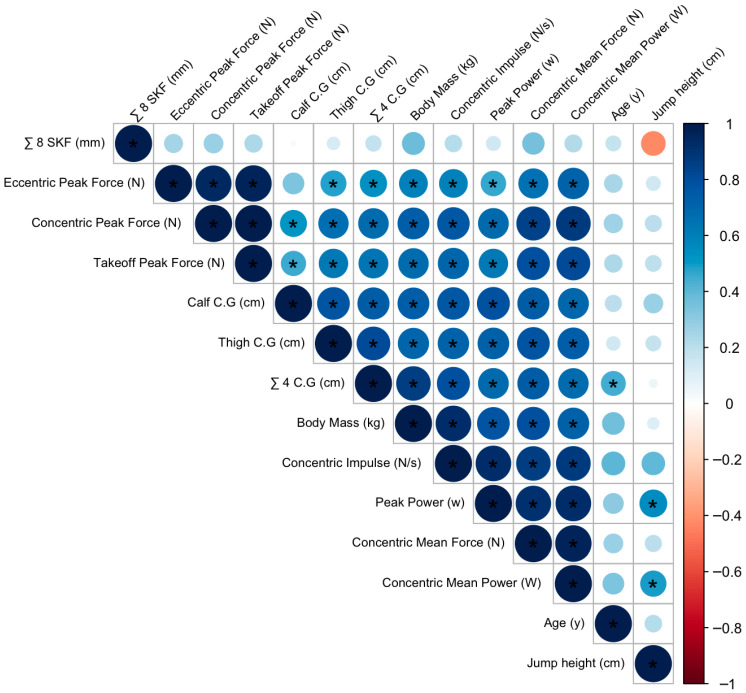
Draftsman correlation plot. Positive correlations are shown in blue and negative correlations in red. Color intensity and circle size are proportional to the correlation coefficients. C.G.: corrected girth; ∑: summation; SKF: skinfolds. * *p* <0.05.

Correlations between anthropometric variables and neuromuscular performance (i.e., CMJ test data) are shown in Figure 2. Strong positive associations between body mass and all neuromuscular performance variables were observed, particularly with concentric impulse (*r* = 0.92; *p* < 0.001) and peak power (*r* = 0.77; *p* < 0.001). Additionally, the corrected calf and thigh girths and the four corrected girths sum exhibited significant positive correlations with most CMJ variables, highlighting the influence of peripheral muscle volume on force and power production. Specifically, the thigh corrected girth showed a positive and high association with concentric mean power (*r* = 0.73; *p* < 0.001) and concentric mean force (*r* = 0.77; *p* < 0.001), while the calf corrected girth had a positive and strong relationship with peak power (*r* = 0.76; *p* < 0.001) and concentric impulse (*r* = 0.76; *p* < 0.001).

Adiposity indicators showed negative correlations with jump height. Specifically, the sum of eight skinfolds (∑8 SKF) displayed a moderate negative correlation (*r* = −0.43), which was noteworthy despite not being statistically significant (*p* > 0.05). This suggests a potential inverse relationship between subcutaneous fat and vertical jump performance, possibly due to the increased inertial load affecting explosive actions.

Finally, stretch stature also showed a positive and strong correlation with concentric impulse (*r* = 0.82; *p* < 0.001).

**Figure 3:**
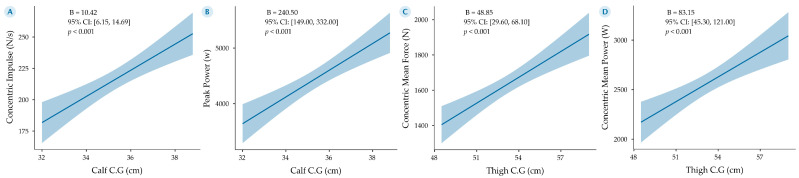
Generalized linear models (GLMs) showing the relationship between corrected girths (calf and thigh) and countermovement jump (CMJ) performance in players from the Mexican National Soccer Team. Models 1A and 1B use corrected calf girth as the main predictor, while Models 1C and 1D use corrected thigh girth. All models were adjusted for body mass, used robust estimation, and assumed a Gaussian distribution. Each panel displays the regression line with its 95% confidence interval (shaded area), alongside the unstandardized regression coefficient (*B*) and corresponding *p*-value. (**A**) Relationship between corrected calf girth and concentric impulse (N·s). (**B**) Relationship between corrected calf girth and peak power output (W). (**C**) Relationship between corrected thigh girth and concentric mean force (N). (**D**) Relationship between corrected thigh girth and concentric mean power (W).

Given the strength and consistency of the observed correlations, particularly those involving corrected girths and neuromuscular outputs, we further explored these relationships through predictive modeling. The results of four generalized linear models (GLMs), aiming to explain CMJ performance based on key anthropometric predictors, are presented in Figure 3.

Model 3A demonstrated a strong positive association between calf corrected girth (Calf C.G) and concentric impulse (N/s), indicating that greater muscle mass in the calf region may enhance force production during the jump. Specifically, each additional centimeter in calf corrected girth was associated with an increase of 10.42 N/s in concentric impulse (*B* = 10.42, 95% CI: [6.15, 14.69], *p* < 0.001).

Model 3B examined peak power (W) as the outcome variable, with calf corrected girth again emerging as a significant predictor. The results showed that each centimeter increase in this corrected girth was associated with an estimated increase of 240.50 W in peak power output (*B* = 240.50, 95% CI: [149.00, 332.00], *p* < 0.001), reinforcing the relevance of lower limb girth in explosive performance.

In Model 3C, thigh corrected girth (Thigh C.G) was significantly associated with concentric mean force, with an increase of 48.85 N per centimeter of thigh girth (*B* = 48.85, 95% CI: [29.60, 68.10], *p* < 0.001). This finding suggests that thigh musculature may also play a crucial role in force development during CMJ.

Finally, Model 3D revealed that thigh corrected girth was positively associated with concentric mean power (W), with each additional centimeter corresponding to an increase of 83.15 W (*B* = 83.15, 95% CI: [45.30, 121.00], *p* < 0.001).

### 3.3. Anthropometric Profile and Players’ Locomotor and Neuromuscular Data

In terms of external load variables derived from GPS, players covered a median distance of 98.8 m/min (IQR: 90.1–105.2), with a maximum speed of 29.8 km/h (IQR: 28.8–30.9). The frequency of accelerations > 3 m/s^2^ was 0.6 per minute (IQR: 0.4–0.7), while decelerations >3 m/s^2^ occurred at a frequency of 0.7 per minute (IQR: 0.5–0.9). The distance covered at high intensity (HMLD) was 16.9 m/min (IQR: 13.8–20.0), with a median of 1.9 sprints per minute (IQR: 1.2–3.0), 0.1 sprint efforts per minute (IQR: 0.1–0.2), and 4.4 m/min of high-speed running (HSR) (IQR: 3.1–6.0). These external load metrics provide a detailed picture of the players’ locomotor performance, which is crucial for understanding their neuromuscular capabilities.

**Figure 4:**
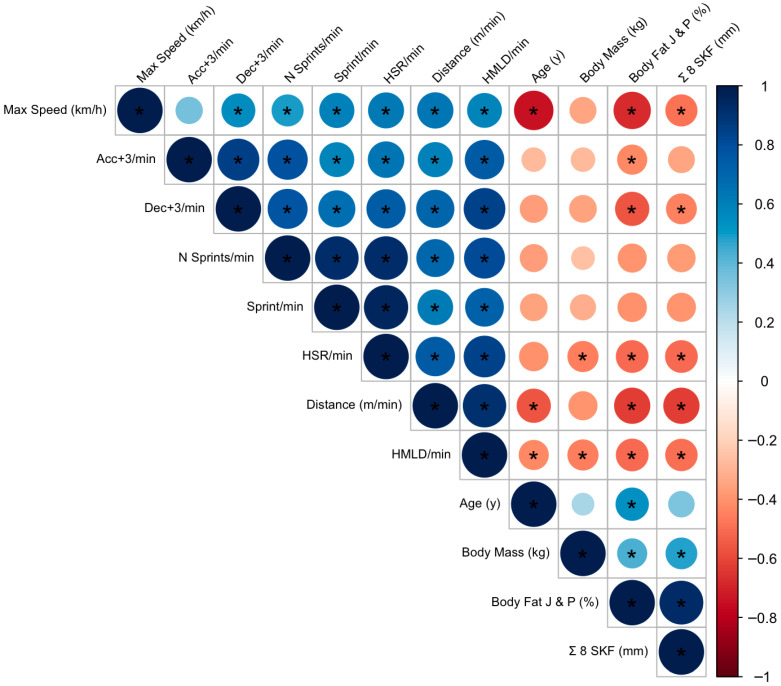
Draftsman correlation plot. Positive correlations are shown in blue and negative correlations in red. Color intensity and circle size are proportional to the correlation coefficients. J and P; Jackson and Pollock’s predictive equation; ∑: summation; SKF: skinfolds. * *p* <0.05.

Principal correlations between anthropometric profile and players’ locomotor and neuromuscular data (i.e., GPS data) are shown in Figure 4. Age demonstrated a consistent and meaningful association with all eight performance variables. Specifically, older players covered less distance per minute (*r* = −0.56; *p* = 0.006), attained lower maximum speed (*r* = −0.67; *p* < 0.001), and accumulated less high metabolic load distance (*r* = −0.75; *p* = 0.001).

Anthropometric indicators of adiposity, namely, the sum of six skinfolds (∑6SKF), the sum of eight skinfolds (∑8SKF), and body fat percentage estimated via Jackson and Pollock’s predictive equation [32] also exhibited relevant correlations. Among these, %BF Jackson displayed the strongest and most consistent inverse associations. For example, a higher %BF was associated with reduced match performance in distance per minute (*r* = −0.63; *p* = 0.001), maximum speed (*r* = −0.62; *p* = 0.002), decelerations (*r* = −0.57; *p* = 0.005), and high-speed running (*r* = −0.53; *p* = 0.011). Similarly, sprint-related metrics such as sprint frequency (*r* = −0.47; *p* = 0.028) and number of sprints (*r* = −0.47; *p* = 0.027) also showed significant negative correlations with %BF.

Although ∑6SKF and ∑8SKF demonstrated slightly weaker associations, notable relationships were still observed. For instance, ∑6SKF was negatively correlated with high-speed running (*r* = −0.52; *p* = 0.012) and HMLD per minute (*r* = −0.49; *p* = 0.021), while ∑8SKF showed similar trends (*r* = −0.52; *p* = 0.012 for both).

**Figure 5:**
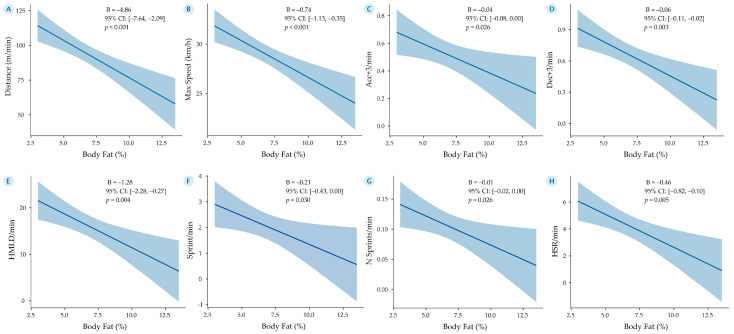
Generalized linear models (GLMs) showing the relationship between body fat percentage (estimated via the Jackson and Pollock equation) and GPS-derived external match load variables in Mexican National Team soccer players. All models included body fat (%) as the main predictor and were fitted using robust estimation and a Gaussian distribution. Each panel displays the regression line with its 95% confidence interval (shaded area), alongside the unstandardized regression coefficient (B) and corresponding *p*-value. (**A**) Distance per minute. (**B**) Maximum speed. (**C**) Number of accelerations > 3 m/s^2^ per minute. (**D**) Number of decelerations > 3 m/s^2^ per minute. (**E**) High metabolic load distance per minute. (**F**) Sprint distance per minute. (**G**) Number of sprints per minute. (**H**) High-speed running per minute.

To further understand the predictive power of body fat on match performance, eight generalized linear models (GLMs) were developed, as illustrated in Figure 5.

Model 5A showed a strong negative association between body fat percentage and in-match distance covered per minute, indicating that higher adiposity may impair players’ locomotor efficiency during a match. Specifically, each 1% increase in body fat was associated with a decrease of 4.86 m per minute (*B* = −4.86, 95% CI: [−7.64, −2.09], *p* < 0.001).

Model 5B examined maximal sprint speed as the outcome variable. Each 1% increase in body fat led to a reduction of 0.74 m/s in sprint speed (*B* = −0.74, 95% CI: [−1.13, −0.35], *p* < 0.001), suggesting that higher adiposity is linked to a lower maximal velocity capacity.

Model 5C focused on high-intensity accelerations (>3 m/s^2^), with each 1% increase in body fat associated with 0.04 fewer accelerations per minute (*B* = −0.04, 95% CI: [−0.08, 0.00], *p* = 0.026).

Model 5D mirrored this trend for decelerations of similar intensity, revealing a decrease of 0.06 per minute for every 1% increase in body fat (*B* = −0.06, 95% CI: [−0.11, −0.02], *p* = 0.003). These results suggest that excessive body fat may hinder the frequency of explosive neuromuscular actions.

Model 5E showed a significant negative relationship between body fat percentage and high metabolic load distance (HMLD) per minute, with a 1.28-m reduction for every 1% increase in fat (*B* = −1.28, 95% CI: [−2.28, −0.27], *p* = 0.004).

Model 5F revealed that the number of sprints per minute declined by 0.21 with each 1% increase in lipid mass (*B* = −0.21, 95% CI: [−0.43, 0.00], *p* = 0.030), reinforcing the limiting impact of adiposity on repeated high-intensity efforts.

Model 5G addressed the total sprint frequency across the match. Each 1% rise in body fat was associated with 0.01 fewer sprints overall (*B* = −0.01, 95% CI: [−0.02, 0.00], *p* = 0.026).

Lastly, model 5H revealed a decline of 0.46 m per minute in high-speed running for each 1% increase in lipid mass (*B* = −0.46, 95% CI: [−0.82, −0.10], *p* = 0.005), further underscoring the detrimental influence of adiposity on repeated sprint and high-velocity actions.

## 4. Discussion

### 4.1. Characteristics of the Anthropometric Profile

The main findings of this study revealed significant differences in physical characteristics across positions, highlighting how these are adapted to the specific demands of each role in the game. Goalkeepers stood out due to their higher body mass, stretch stature, and girths, reflecting their need for stability and control in physical contact situations. Additionally, this group exhibited the highest body fat percentages and estimated muscle mass, attributes linked to the demands of their position. In contrast, offensive midfielders were characterized by lower body mass, stretch stature, skinfold thickness, and girths. This profile aligns with their tactical role, which requires high agility and dynamism.

The lateral and central defenders showed specific positional differences in body mass and girths, reflecting physical adaptations related to their contact and coverage functions.

A comparison between the results of this study and an anthropometric study conducted on Mexican national team players who participated in the 2002 Korea/Japan World Cup [4] highlighted the following points of comparison. The players in this study had higher average values for body mass (77.9 vs. 74.0 kg) and stretch stature (179.4 vs. 175.0 cm). Regarding body composition, the footballers in this study showed a lower body fat percentage (5.9 vs. 10.0%) compared to those evaluated 23 years ago in the pre-competitive stage leading up to the aforementioned World Cup. It is important to note that the average estimate for body fat percentage in the 2002 sample was derived from the same Jackson and Pollock equation used in this study. Therefore, based on an average extrapolation, current players have higher muscle mass and lean body mass, with an average of 3.9 kg more body mass.

The differences between the previous study and this one were significant. The previous research did not differentiate playing positions, while the current study clearly distinguishes physical characteristics by position, also incorporating specific indicators such as corrected thigh and leg girths, which emphasize the importance of peripheral muscle development in locomotor performance.

Most of the publications describing the anthropometric profile of soccer players at the international level have focused on players from lower categories at the state level [5] or from the first division in different countries [6,7,8,9,43,44]. Compared to 184 Italian players from Serie A studied by Petri et al. [9], the Mexican players in this sample had a shorter stretch stature (Mex: 179.9 cm vs. Ita: 183.9 cm), lower average body mass (Mex: 76.8 kg vs. Ita: 79.2 kg), a lower sum of eight skinfolds (Mex: 51.5 mm vs. Ita: 59 mm), and a similar BMI (Mex: 23.6 kg/m^2^ vs. Ita: 23.3 kg/m^2^).

In comparison to first division players from Brazil and Argentina, the Mexican soccer players showed similar characteristics in terms of average body mass and stretch stature for each playing position [7,8]. Qualitatively, goalkeepers consistently had higher body mass and stretch stature than other positions, while offensive midfielders showed the lowest values. Quantitatively, the Mexican goalkeepers in the sample analyzed (186.2 cm and 84.5 kg) were in the intermediate range compared to Brazilian goalkeepers (188.1 cm and 88.7 kg) and Argentine goalkeepers (185.4 cm and 83.7 kg). Similarly, the Mexican offensive midfielders (173.5 cm and 68.6 kg) had morphological characteristics similar to those of Brazilian players (176.4 cm and 71.9 kg) and Argentine players (173.2 cm and 71.6 kg). These findings suggest that, while there are slight differences between countries, the anthropometric dimensions of Mexican soccer players do not differ substantially from those reported in the leading South American leagues.

However, despite the clear similarities between the players studied and those from these two countries, football is one of the sports with the greatest morphological variability at the international level, even within each playing position. While there are certain trends within positions according to their function, the variability within each is considerably wide [45]. For example, among the players who participated in the 2018 World Cup in Russia, goalkeepers had stretch stature and body mass variability ranges from 178 to 201 cm and 70 to 98 kg, defenders from 167 to 200 cm and 61 to 99 kg, midfielders from 165 to 197 cm and 59 to 90 kg, and forwards from 168 to 197 cm and 61 to 98 kg. This suggests that anthropometric demands may not contribute significantly to the variance associated with success, as occurs in sports like basketball, which is more dependent on absolute stretch stature [46].

The sum of skinfolds was another variable in which Mexican players showed lower average values (38.5 mm) compared to Argentine footballers (50.9 mm), considering the sum of six skinfolds in both cases. Similarly, when analyzing the sum of eight skinfolds, the Mexican players’ averages (50.5 mm) were lower than those reported for Italian players (51.3 mm), all belonging to first division teams in their respective countries. It is worth mentioning that this research not only presents the sum of skinfolds but also provides reference values obtained through various equations for estimating body fat percentage, which will allow for broader comparisons with other populations of footballers in future studies. This characteristic constitutes one of the strengths of this work [20,21,22,23,24,25,26,27,28,29,30].

Most of the internationally known equations in the sports field have been validated in different countries using DXA, although not all studies have yielded the same results. This highlights the principle of population specificity and the need to employ various equations in the study of population groups, as in this research [47,48,49]. In Mexico, the equations by Oliver, Ball, Civar, and Wilmore were the most accurate for estimating body fat percentage (BF%) compared to DXA in male professional footballers [47]. In Colombia, the body fat percentage calculation using the five-site split and the Durnin and Womersley equation most closely matched the results obtained with DXA in professional Colombian soccer players [48]. In Spanish first division players, the best field equation for assessing body fat percentage was based on iliac crest and triceps skinfolds, as well as the sum of triceps, subscapular, suprailiac, and abdominal skinfolds, since this combination also showed a good correlation with DXA [49].

### 4.2. Associations Between Anthropometric Profile and Neuromuscular Performance

Neuromuscular performance, particularly in tasks requiring explosiveness such as jumping, has been widely investigated in sports science. A notable example is the study by Gutiérrez and Monrroy [4], who assessed the Mexican national soccer team prior to their participation in the FIFA World Cup Korea/Japan 2002, reporting an average vertical jump height of 45 ± 8 cm. This finding highlights the relevance of explosive capacities in elite-level soccer players.

In line with this approach, the present study also evaluated neuromuscular performance in a sample of 22 soccer players using the countermovement jump (CMJ) test, yielding an average jump height of 41.65 ± 3.41 cm. It is worth noting that, due to methodological differences between tests, the values obtained via CMJ may have been higher if the traditional vertical jump protocol used in 2002 had been applied.

Beyond jump height, this study adopted a more integrative perspective of neuromuscular performance by identifying significant associations between corrected muscle girths and strength and power variables such as concentric impulse (*r* = 0.92; *p* < 0.001) and peak power (*r* = 0.77; *p* < 0.001). These results are consistent with an association between muscular development and explosive performance.

These findings align with the observations of Ramírez-Munera et al. [13], who highlighted that greater muscle volume significantly enhances performance in explosive actions. Additionally, a negative correlation was observed between the sum of eight skinfolds (Σ8 Skinfolds) and jump height (*r* = −0.43), which, although not statistically significant, provides theoretical evidence of the potential impact of adiposity on neuromuscular performance.

This result is consistent with the hypothesis that a higher lipid mass may be associated with an increased inertial load, as noted by authors such as Toro-Román et al. [11]. However, the lack of statistical significance may be due to the limited sample size or the need for more position-specific analyses.

Furthermore, a positive correlation was identified between stretch stature and concentric impulse (*r* = 0.82; *p* < 0.01), suggesting that players with greater limb length may benefit from more favorable mechanics to generate force during take-off. This association is consistent with the findings reported by Bustamante-Garrido et al. [9], reinforcing the link between biomechanics and performance in disciplines that demand explosive abilities.

### 4.3. Associations Between the Anthropometric Profile and Locomotor Performance

Body fat percentage (%BF) is associated with differences in the locomotor performance of soccer players, particularly when considering metrics obtained through GPS monitoring. In this study, significant negative associations were identified between %BF and key variables such as distance covered per minute (*B* = −4.86, *p* < 0.001) and maximum sprint speed (*B* = −0.74, *p* < 0.001). These results indicate that higher levels of adiposity are associated with reduced locomotor efficiency and, consequently, the players’ tactical responsiveness during match play.

These findings are consistent with those reported by Enes et al. [50], who concluded that a lower proportion of body fat, together with better aerobic system development, is associated with superior sprint performance—underscoring the importance of the anthropometric profile in meeting the demands of modern soccer.

Additionally, it was observed that an increase in %BF was associated with a reduced ability to perform accelerations above 3 m/s^2^ (*B* = −0.04, *p* = 0.026) and rapid decelerations (*B* = −0.06, *p* = 0.003). These results support the findings of Daly et al. [51], who reported that players with lower body fat percentages were able to sustain a higher number of high-intensity sprints during a match.

From a tactical standpoint, these limitations are especially relevant for positions such as wingers or forwards, who rely on explosive actions to gain advantages in tight spaces or rapid transitions. Consequently, body composition management emerges not only as a matter of health and fitness but also as a strategic tool for optimizing soccer performance.

### 4.4. Relevance of the Mathematical Model and Comparison with Other Approaches

The implementation of generalized linear models (GLMs) with robust estimation and adjustment for key variables such as body mass and age represents one of the main methodological contributions of this study. This approach not only reduces the influence of outliers and heteroscedasticity issues but also ensures that the observed associations are statistically robust and representative of the specific context of the players evaluated.

Unlike previous approaches, such as that proposed by Fields et al. [52], which incorporate mixed metrics or lack individualized adjustments, the model used in this study focuses exclusively on objective and standardized variables. This provides a framework for exploring associations between adiposity indicators (Σ8 skinfolds and %BF) and specific locomotor metrics such as HMLD/min and HSR/min. This approach yielded findings of high practical utility for monitoring and intervention in professional sports contexts.

Adjusting for age and body mass provides the model with a particular sensitivity to detect differences within a restricted and homogeneous group, such as the Mexican national team. In contrast to broader but less personalized studies, such as that of Daly et al. [50], this methodology enables the extraction of relevant individual-level patterns and the generation of specific recommendations for designing physical and nutritional interventions.

The results show that body fat percentage (%BF) has a significant negative association with various locomotor metrics. For example, the distance covered per minute yielded a coefficient of *B* = −4.86 (95% CI: [−7.64, −2.09], *p* < 0.001), indicating that, for each additional unit of %BF, players cover, on average, 4.86 m per minute. This finding underscores how higher adiposity levels compromise movement efficiency and are associated with differences in player involvement in offensive and defensive transitions.

Previous studies support this association. Varley et al. [53], for instance, reported that increased leg cell mass positively correlates with metrics such as total distance covered (*r* = 0.44), accelerations (*r* = 0.45), and decelerations (*r* = 0.49), suggesting that body composition is associated with differences in locomotor efficiency. Similarly, Şahin and Sanioğlu [54], in their analysis of 63 amateur Turkish footballers, observed that increases in %BF negatively affected 10- and 15-m sprint distances and acceleration, explaining up to 9.4% of the variance in performance.

From a tactical perspective, these locomotor limitations have clear implications. Positions such as wingers and attacking midfielders require a high capacity for sustained movement to generate passing lanes, support transitions, and apply high pressure. An elevated %BF may restrict these functions, reducing the player’s tactical effectiveness. In this regard, Kapteijns et al. [55] emphasized that soccer players with more efficient body composition profiles tend to perform a greater number of high-intensity actions, particularly in roles that demand constant dynamism.

Additionally, the study showed that a higher %BF is associated with lower maximum sprint speed (*B* = −0.74, 95% CI: [−1.13, −0.35], *p* < 0.001), which is associated with a reduced ability to compete in one-on-one duels or recover defensive positions. These results are consistent with those of Ishida et al. [12], who found significant correlations between %BF and 10-m sprint times (*r* = −0.45 to −0.53) and 20-m sprint times (*r* = −0.40 to −0.49), reinforcing the notion that body fat is a limiting factor in explosive soccer performance.

### 4.5. Strengths and Limitations

This study presents several methodological and analytical strengths that enhance the validity of its findings. First, internationally validated methods were used to estimate body composition, including specific equations and comparative metrics according to playing position. This methodological diversity enables a more accurate, contextualized, and applicable assessment across different player profiles within soccer. Additionally, the consideration of anthropometric differences by playing position provides relevant insights into the specific physical demands imposed by each role on the field, which can inform both training program design and talent identification and development processes.

Another notable strength lies in the statistical robustness of the analytical approach employed. The mathematical model is based on generalized linear models (GLMs) with robust estimation controls for the influence of outliers and heteroscedasticity, thereby increasing the reliability of the observed associations. This type of analysis facilitates the identification of consistent and clinically meaningful patterns, even in moderate sample sizes such as the one used in this study.

However, certain limitations should also be acknowledged. The relatively small sample size and its restriction to Mexican players limit the generalizability of the results to other populations or competitive levels. This means that, although the findings are robust within the specific context analyzed, caution is required when extrapolating them to different settings, such as European leagues or youth categories. Moreover, the exclusion of interaction terms from the model limits the exploration of potential combined effects between key variables, such as the interaction between playing position and body fat percentage. This limitation reduces the model’s capacity to capture the inherent complexity of soccer performance.

Lastly, the cross-sectional nature of the study prevents the observation of longitudinal changes in anthropometric profiles and performance over time. Incorporating a longitudinal analysis would enable a more comprehensive evaluation of the effects of training, competitive load, and other contextual variables on body composition and locomotor metrics.

### 4.6. Practical Implications

The models developed in this study suggest that optimal body composition—particularly, lower adiposity and well-developed lower limb musculature—is associated with enhanced locomotor and neuromuscular performance in soccer.

This finding highlights the importance of implementing individualized training and nutritional strategies tailored to the specific demands of each playing position. Furthermore, the use of generalized linear models (GLMs) allowed for a clear identification of these relationships, reinforcing the value of regular body composition monitoring as a potential indicator for tailoring strategies to support physical and tactical performance.

## 5. Conclusions

This study provides solid evidence of the influence of body composition on the athletic performance of soccer players. The results highlight position-specific differences in the anthropometric profiles of professional players. Furthermore, this exploratory analysis identified general associations between adiposity, GPS-derived metrics, and neuromuscular capacity. Although these associations were not examined by playing position due to sample size limitations, they provide valuable insights for performance monitoring and optimization. The findings emphasize the importance of tailoring dietary, training, and strategic programs to positional and tactical demands, thereby supporting a comprehensive approach to maximize individual efficiency and performance.

## Figures and Tables

**Table 1:** Anthropometric characteristics of Mexican National Team soccer players for the overall sample and by playing position.

Variables	Goalkeepers (*n* = 4)	Lateral (Outside) Defenders (*n* = 5)	Central Defenders (*n* = 6)	Offensive Midfielders (*n* = 7)	Defensive Midfielders (*n* = 6)	Forwards (*n* = 8)	Overall Sample (*n* = 36)
	Median (IQR)	Median (IQR)	Median (IQR)	Median (IQR)	Median (IQR)	Median (IQR)	Median (IQR)
Age (y)	26.6 (24.2–31.0)	27.3 (27.2–27.9)	25.7 (23.6–27.2)	26.5 (24.8–27.7)	27.0 (24.2–29.4)	29.1 (24.2–32.8)	27.3 (24.2–28.4)
**Basics**							
Body Mass (kg)	83.9 * (83.5–84.9)	75.0 (72.7–76.3)	82.8 † (79.2–83.9)	69.7 *† (63.8–70.9)	74.0 (72.4–78.0)	79.5 (76.5–81.7)	77.9 (71.3–82.9)
Stretch stature (cm)	186.2 * (182.6–189.7)	177.5 (176.5–177.9)	185.7 † (183.0–189.0)	174 *† (169.5–175.1)	181.7 (177.8–184)	179.1 (176.0–184.9)	179.4 (175.4–184)
**Skinfolds (mm)**							
Triceps	7.1 (5.9–8.8)	6.0 (4.0–9.0)	6.0 (5.0–7.0)	5.0 (4.0–5.2)	6.5 (5.8–7.0)	5.5 (4.8–6.3)	5.6 (5.0–7.0)
Subscapular	7.1 (6.8–8.9)	8.0 (7.0–9.0)	7.5 (6.3–8.0)	7.2 (6.2–8.0)	8.5 (7.5–9.3)	8.0 (7.0–9.0)	8.0 (6.8–8.3)
Biceps	4.0 (3.8–4.0)	3.0 (3.0–4.0)	3.5 (3.0–4.0)	3.0 (3.0–3.0)	4.0 (3.8–4.3)	3.0 (3.0–3.0)	3.0 (3.0–4.0)
Iliac crest	15.0 (10.3–19.3)	8.0 (7.0–9.0)	9.5 (6.8–10.8)	8.0 (7.0–11.0)	10.0 (8.8–11.0)	9.0 (6.8–11.5)	9.0 (7.0–11.0)
Supraspinal	7.0 (6.6–8.0)	5.0 (4.0–6.0)	4.0 (3.3–5.5)	5.0 (4.0–6.0)	5.0 (4.0–5.3)	5.0 (4.8–5.2)	5.0 (4.0–6.0)
Abdominal	15.0 (11.1–18.3)	8.0 (5.8–11.0)	7.5 (6.3–8.8)	8.0 (7.0–10.2)	10.0 (9.5–11.3)	9.0 (8.0–11.0)	8.7 (7.0–11.0)
Thigh	6.2 (5.8–7.1)	6.0 (4.4–6.0)	6.0 (5.3–6.8)	5.0 (4.0–6.0)	5.5 (4.8–6.3)	5.0 (4.8–5.3)	6.0 (5.0–6.3)
Calf	4.0 (3.8–4.2)	3.2 (3.0–6.0)	5.0 (3.5–5.0)	3.2 (3.0–4.0)	6.3 (5.5–6.3)	8.5 (7.1–11.0)	4.0 (3.0–5.0)
Chest	8.7 (6.3–11.5)	5.0 (5.0–6.8)	5.0 (5.0–5.8)	6.0 (4.0–8.0)	5.5 (4.8–6.3)	6.0 (5.8–7.0)	6.0 (5.0–7.0)
**Girths (cm)**							
Arm (relaxed)	33.3 (32.1–34.3)	32.0 (31.5–32.5)	32.8 (32.1–33.8)	31.0 (30.5–32.0)	27.0 (26.9–27.5)	32.5 (32.0–33.1)	32.0 (31.0–33.0)
Forearm	28.8 * (28.4–29.0)	28.0 (27.5–28.2)	28.0 (27.6–28.4)	26.5 * (26.0–27.0)	27.0 (26.9–27.5)	27.8 (26.9–28.1)	27.5 (27.0–28.3)
Chest	104.0 (101.3–106.3)	100.0 (100.0–100.0)	102.8 (101.5–104.8)	98.0 (95.0–100.5)	95.3 (94.4–98.1)	103.5 (101.5–104.0)	100.8 (98.0–104.0)
Thigh (middle)	55.0 (54.9–55.4)	54.2 (53.5–55.5)	56.8 (54.9–58.6)	53.5 (52.5–54.0)	55.5 (54.3–56.4)	55.8 (55.2–58.3)	55.0 (53.9–56.1)
Calf	36.7 (36.4–37.2)	34.5 (34.5–37.0)	37.9 † (37.1–39.2)	35.2 †‡ (34.5–36.0)	36.0 (35.4–37.0)	39.0 ‡ (37.8–39.0)	37.0 (35.5–38.3)
**Indexes**							
BMI (kg/m^2^)	24.4 (23.9–24.9)	24.1 (23.1–24.2)	23.3 (23.3–24.0)	22.4 (22.2–23.0)	23.5 (22.7–24.3)	23.9 (23.9–24.8)	23.8 (23.0–24.6)
HWR	42.6 (42.0–43.2)	42 (41.8–42.6)	43.2 (42.4–43.4)	42.4 (42.3–42.9)	42.2 (41.7–43.1)	42.4 (41.6–42.7)	42.4 (41.8–43.0)
**Sum of skinfolds (mm)**							
Σ 6 skinfolds §	48.0 (38.8–57.8)	37 (29.6–44.0)	36.5 (31.3–41.0)	37 (28.4–40.0)	40 (37.3–42.8)	37 (33.3–44.2)	38.5 (30.8–43.4)
Σ 8 skinfolds #	67.5 (52.8–81.5)	48.0 (39.6–57.0)	50.0 (41.3–55.0)	48.0 (38.0–54.2)	53.5 (50.8–56.5)	49 (43.0–59.7)	50.5 (39.9–59.1)
**Body fat** **lipid tissue mass (%)**							
Durnin and Womersley, 1974	15.4 (12.3–18.7)	10.4 (9.9–14.6)	12.1 (10.1–13.5)	11.0 (9.1–12.4)	13.4 (12.1–14.4)	11.5 (9.5–14.4)	12.3 (9.8–14.2)
Sloan, Burt, and Blyth, 1962	6.9 (6.3–7.8)	6.4 (5.7–7.2)	6.5 (5.8–6.7)	6.2 (5.1–6.7)	6.7 (6.3–7.0)	6.5 (5.9–7.9)	6.5 (5.5–7.2)
Wilmore and Behnke, 1969	12.7 (11.1–14.1)	10.1 (9.0–11.2)	10.1 (9.3–10.6)	10.1 (9.3–10.9)	10.7 (10.4–11.3)	10.3 (9.6–11.4)	10.5 (9.5–11.3)
Jackson and Pollock, 1980	8.4 (6.1–11.2)	5.3 (4.2–6.6)	5.5 (4.2–6.2)	6.3 (3.9–6.3)	6.3 (5.4–7.0)	5.4 (5.3–7.1)	5.9 (4.6–6.7)
Yuhasz, 1961	10.9 (9.5–12.3)	9.2 (8.5–10.0)	9.4 (8.6–9.9)	9.1 (8.3–9.8)	9.8 (9.5–10.0)	9.4 (8.8–10.4)	9.6 (8.5–10.3)
**Adipose tissue mass (%)**							
Ross and Kerr, 1991	20.5 (19.3–22.0)	17.9 (16.9–19.1)	18.2 (17.0–19.7)	18.4 (17.1–20.0)	19.4 (19.0–19.5)	18.0 (16.3–20.0)	18.8 (17.0–20.0)
**Corrected girths (cm)**							
Arm C.G	31.2 (30.1–31.8)	30.0 (29.7–30.2)	31.4 (30.5–31.8)	29.4 (28.6–30.7)	29.4 (28.8–30.2)	30.4 (29.8–32.0)	30.2 (29.5–31.3)
Chest C.G	100.7 (99.1–102.5)	97.8 (97.2–98.1)	100.6 (99.2–102.5)	95.7 (93.1–98.3)	92.8 (92.0–95.5)	101.2 (98.3–101.6)	98.4 (95.7–101.4)
Thigh C.G	53.3 (52.9–53.5)	52.9 (52.1–53.6)	54.9 (53.0–56.7)	51.6 (49.8–52.7)	53.8 (52.4–54.7)	54.1 (53.7–55.5)	53.5 (52.3–54.2)
Calf C.G	35.3 (35.2–35.9)	33.5 (33.1–36.1)	36.6 † (35.5–38.1)	33.9 †‡ (33.7–34.6)	35.7 (35.6–36.2)	37.7 ‡ (36.4–37.8)	35.4 (34.3–37.2)
Σ4 C.G	221.3 (217.8–224.0)	216.3 (214.5–217.4)	223.2 † (220.5–225.6)	209.5 †‡ (207.2–214.7)	211.4 (209.8–215.4)	222.2 ‡ (219.4–227.3)	217.8 (212.0–222.6)
**Muscle mass (kg)**							
Martin, et al., 1990	49.4 (49.2–49.8)	45.0 (43.6–47.5)	52.4 † (49.4–53.4)	42.6 †‡ (39.1–43.4)	45.5 (44.8–47.7)	49.4 ‡ (46.7–51.7)	47.5 (43.6–50.0)
Lee, et al., 2000	37.6 (36.8–37.9)	34.2 (34.0–35.0)	39.2 † (37.1–39.8)	32.6 †‡ (32.4–34.7)	34.7 (34.2–36.0)	38.2 ‡ (36.5–38.7)	35.1 (34.0–38.3)

Data are presented as the median (IQR). Statistically significant differences (adjusted *p* < 0.05) were assessed with Dunn’s post hoc test (Bonferroni correction). Symbols (*, †, and ‡) denote significant differences between groups: * between goalkeepers and offensive midfielders, † between central defenders and offensive midfielders, and ‡ between offensive midfielders and forwards. § Sum of triceps, subscapular, supraspinal, abdominal, thigh, and calf skinfolds. # Sum of triceps, subscapular, biceps, iliac crest, supraspinal, abdominal, thigh, and calf skinfolds. Abbreviations: C.G, correct girth; HWR, height-to-weight ratio; BMI, body mass index.

## Data Availability

The original contributions presented in this study are included in the article. Further inquiries can be directed to the corresponding author(s).

## Abbreviations

The following abbreviations are used in this manuscript:

ISAK	International Society for the Advancement of Kinanthropometry
CMJ	Countermovement Jump
GPS	Global Positioning System
DXA	Dual-Energy X-ray Absorptiometry
GLM	Generalized Linear Model
%BF	Percent Body Fat
MM	Muscle Mass
HSR	High-Speed Running
HMLD	High Metabolic Load Distance
TD	Total Distance
